# Chemical Composition of *Kickxia aegyptiaca* Essential Oil and Its Potential Antioxidant and Antimicrobial Activities

**DOI:** 10.3390/plants11050594

**Published:** 2022-02-23

**Authors:** Ahmed M. Abd-ElGawad, Yasser A. El-Amier, Giuliano Bonanomi, Abd El-Nasser G. El Gendy, Abdallah M. Elgorban, Salman F. Alamery, Abdelsamed I. Elshamy

**Affiliations:** 1 Department of Botany, Faculty of Science, Mansoura University, Mansoura 35516, Egypt; yasran@mans.edu.eg; 2 Department of Agriculture, University of Naples Federico II, 80055 Naples, Italy; giuliano.bonanomi@unina.it; 3 Department of Medicinal and Aromatic Plants, National Research Centre, Dokki, Cairo 12311, Egypt; aggundy_5@yahoo.com; 4 Department of Botany and Microbiology, College of Science, King Saud University, Riyadh 11451, Saudi Arabia; aelgorban@ksu.edu.sa; 5 Biochemistry Department, College of Science, King Saud University, Riyadh 11451, Saudi Arabia; salamery@ksu.edu.sa; 6 Department of Natural Compounds Chemistry, National Research Centre, 33 El Bohouth St., Dokki, Giza 12622, Egypt; elshamynrc@yahoo.com

**Keywords:** volatile oils, *Linaria aegyptiaca*, biological activity, cuminic aldehyde, sesquiterpenes

## Abstract

The exploration of new bioactive compounds from natural resources as alternatives to synthetic chemicals has recently attracted the attention of scientists and researchers. To our knowledge, the essential oil (EO) of *Kickxia aegyptiaca* has not yet been explored. Thus, the present study was designed to explore the EO chemical profile of *K. aegyptiaca* for the first time, as well as evaluate its antioxidant and antibacterial activities, particularly the extracts of this plant that have been reported to possess various biological activities. The EO was extracted from the aerial parts via hydrodistillation and then characterized by gas chromatography-mass spectrometry (GC-MS). The extracted EO was tested for its antioxidant activity via the reduction in the free radicals, 2,2-diphenyl-1-picrylhydrazyl (DPPH) and 2,2′-azinobis(3-ethylbenzothiazoline-6-sulfonic acid) (ABTS). In addition, the EO was tested as an antibacterial mediator against eight Gram-negative and Gram-positive bacterial isolates. Forty-three compounds were identified in the EO of *K. aegyptiaca*, with a predominance of terpenoids (75.46%). Oxygenated compounds were the main class, with oxygenated sesquiterpenes attaining 40.42% of the EO total mass, while the oxygenated monoterpenes comprised 29.82%. The major compounds were cuminic aldehyde (21.99%), caryophyllene oxide (17.34%), hexahydrofarnesyl acetone (11.74%), *ar*-turmerone (8.51%), aromadendrene oxide (3.74%), and humulene epoxide (2.70%). According to the IC_50_ data, the *K. aegyptiaca* EO revealed considerable antioxidant activity, with IC_50_ values of 30.48 mg L^−1^ and 35.01 mg L^−1^ for DPPH and ABTS, respectively. In addition, the EO of *K. aegyptiaca* showed more substantial antibacterial activity against Gram-positive bacterial isolates compared to Gram-negative. Based on the minimum inhibitory concentration (MIC), the EO showed the highest activity against *Escherichia coli* and *Bacillus cereus*, with an MIC value of 0.031 mg mL^−^^1^. The present study showed, for the first time, that the EO of *K. aegyptiaca* has more oxygenated compounds with substantial antioxidant and antibacterial activities. This activity could be attributed to the effect of the main compounds, either singular or synergistic. Thus, further studies are recommended to characterize the major compounds, either alone or in combination as antioxidants or antimicrobial agents, and evaluate their biosafety.

## 1. Introduction

Human beings are putting increased pressure on the planet’s various resources. Scientists and researchers are doing their best to explore new, green, eco-friendly natural bioactive compounds that can be used in various treatments in agriculture, pharmaceuticals, and industry [1]. Plants are the main source of bioactive compounds (phytochemicals). Essential oils (EOs) are considered promising bioactive compounds due to their various biological activities and their chemical diversity [2,3,4]. *Kickxia* genus includes 47 species worldwide including Africa, Europe, Asia, and Macaronesia [5]. The *Kickxia* genus is one of the largest genera of the family Plantaginaceae in the flora of Egypt, where it is represented by 11 species [6]. The plants belonging to *Kickxia* genus are well known for the presence of several metabolites such as flavonoids [7,8], alkaloids [9], terpenoids [10], and iridoids [11]. Several traditional uses of *Kickxia* species around the world have been documented, such as laxatives, diuretics, tonics, anti-diabetic, and antiscorbutic, alongside the treatment of disorders such as hemorrhoids, wounds, and vascular treatments [12].

*Kickxia aegyptiaca* (L.) Nábělek is a wild perennial herbal plant of the family Plantaginaceae. It is widely distributed in Egyptian sandy plains, wadis, deserts, Sinai Peninsula, oases, and the Mediterranean coastal areas [13]. Its synonyms include *Antirrhinum aegyptiacum*, *Linaria micromerioides*, and *Linaria aegyptiaca* [6]. The plant grows up to 50 cm, with a dense and woody base. Leaves are ovate or lanceolate, with an entire or dentate margin. The plant has yellow flowers, and flowering time extends from February to June [13]. *Kickxia* species, including *K. aegyptiaca,* were documented as significant traditional plants in the treatment of vascular diseases, haemorrhoids, and wounds, along with their uses as laxatives, anti-diabetics, anti-scorbutics, diuretics, and, tonics agents [12,14]. The phytochemical characterization of this plant revealed that it is rich in flavonoids, phenolic acids, glycosides, and iridoids [7,15,16]. The different *K. aegyptiaca* extracts and their isolated metabolites were documented to have antioxidant activity [17,18], larvicidal activity [18], and cytotoxic activity [15,19]. Flavonoids, pectolinarigenin, tangeretin, and gardenin were also isolated from *K. aegyptiaca,* which exhibited potential antiviral activity against SARS-CoV-2 [16].

To our knowledge, and according to the literature review, only *K.* *spuria* EO chemical profile was described by Morteza-Semnani, Saeedi and Akbarzadeh [10]. However, its EO *K. aegyptiaca* has not been studied to date to the best of our knowledge. Therefore, the present document (i) describes, for the first time, the chemical characterization of EO of the aerial parts of *K. aegyptiaca*, and (ii) evaluates the antioxidant and antibacterial potential of its EO.

## 2. Results and Discussion

### 2.1. Chemical Characterization of K. aegyptiaca EO

**Figure 1:**
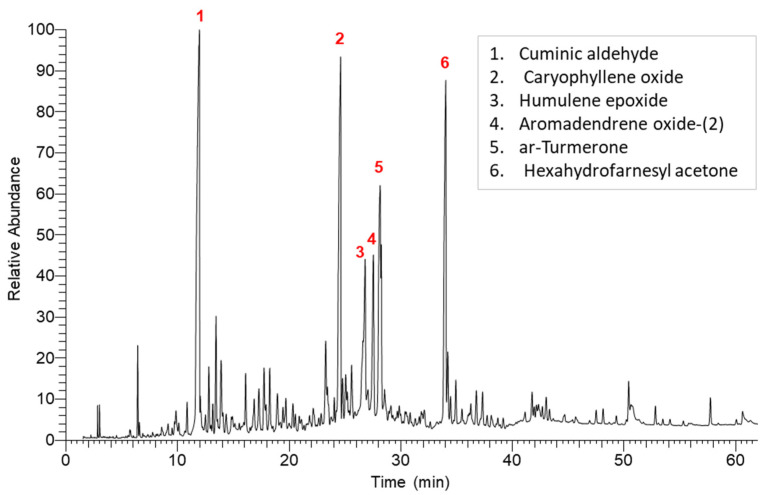
Chromatogram of *K. aegyptiaca* EO compounds derived from GC-MS analysis. The peaks in major compounds are numbered (1–6).

The hydrodistillation of *K. aegyptiaca* aerial parts produced a golden–yellow oil of 0.51% (*v*/*w*). This amount of EO was found to be comparable to that obtained from *K. spuria*, 0.40% [10]. The extracted EO was analyzed by GC-MS and the ion chromatogram is presented in Figure 1. The chemical characterization led to the identification of 43 components, which are comparable in number with those identified in the EO of *K. spuria* [10]. The identified compounds represented 97.36% of the extracted EO.

**Figure 2:**
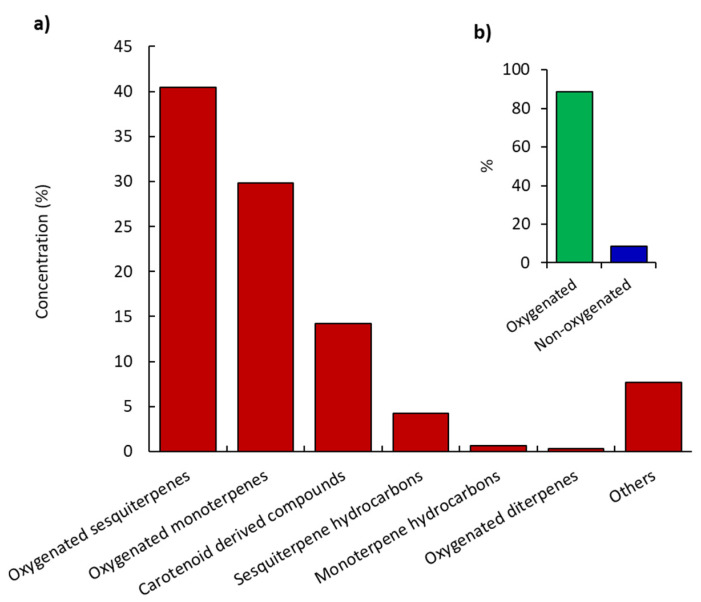
Percentage of the various classes of the recognized chemical compounds (**a**) and the total oxygenated and non-oxygenated compounds (**b**) in the essential oil of the *K. aegyptiaca*.

The identified chemical compounds are listed in detail, along with their retention times (Rt), and Kovats indexes (KI) in Table 1. These compounds can be categorized into seven classes of components, including monoterpenes (oxygenated and hydrocarbons), sesquiterpenes (oxygenated and hydrocarbons), diterpenes (oxygenated only), carotenoid derived components, and other hydrocarbons (Figure 2). These data revealed that this oil is very rich in terpenoid compounds, which represented 75.46% of the total EO mass. From overall terpenoids, a relative concentration of 70.57% was found in oxygenated forms. The abundance of terpenoids, particularly the oxygenated ones, was in harmony with the published data for *K. spuria* EO [10].

**Figure 3:**
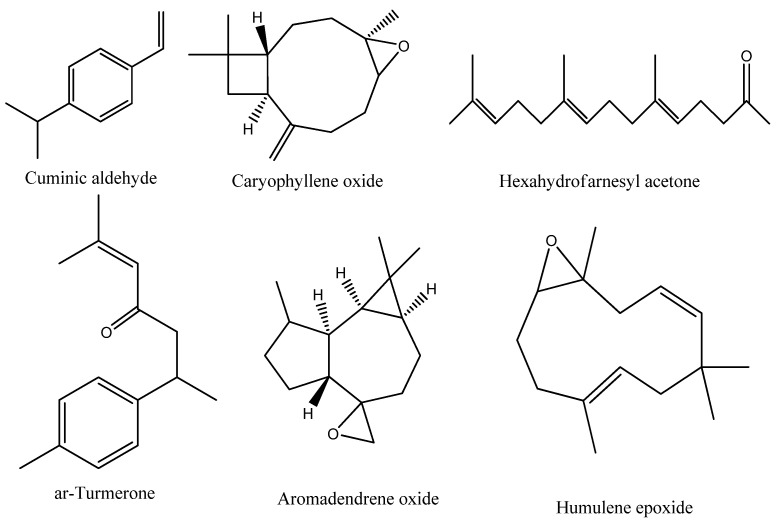
Chemical structure of the major identified compounds in the EO of *K. aegyptiaca*.

Sesquiterpenes were identified with a relative concentration of 44.68% of the overall oil mass, including both the oxygenated sesquiterpenes (40.42%) and sesquiterpenes hydrocarbon (4.26%) forms. The dominance of the sesquiterpenes is also described in the EO of *K. spuria* [10]. Out of the 14 identified oxygenated sesquiterpenes, caryophyllene oxide (17.34%), *ar*-turmerone (8.51%), aromadendrene oxide (3.84%), and humulene epoxide (2.70%) represented the major ones (Figure 3), while *trans*-nerolidol (0.25%) represented the minor.

Caryophyllene oxide was determined in a high concentration (8.90%) in the EO of *K. spuria* [10]. Some of the identified compounds were also described in the constituents of *K. spuria* EO, such as *ar*-curcumene, spathulenol, as well as the *cis* isomer of sesquisabinene hydrate. Caryophyllene oxide, *ar*-turmerone, aromadendrene oxide, and humulene epoxide are widely distributed compounds in the EOs of several plants, such as *Centaurea* species [20], *Artemisia campestris* [21], *Cullen plicata* [2], *Chromolaena odorata* [22], and *Heliotropium curassavicum* [23]. On the other side, five sesquiterpene hydrocarbons were assigned, including isocaryophillene (1.97%) and longicyclene (1.07%) as the main compounds.

Monoterpenes represented the second class of identified compounds (30.45%), which encompass oxygenated monoterpenes as the main compounds, with a relative concentration of 29.82%, along with 0.63% of monoterpene hydrocarbons. Seven compounds were assigned as oxygenated monoterpenes, in which cuminic aldehyde (21.99%) and *p*-cymen-7-ol (2.17%) were determined as major compounds. The profile of the monoterpenes was totally different compared to that reported in *K. spuria* EO, except for eugenol [10]. This variation could be ascribed to the genetic differences in both species [24], and the environmental and climatic conditions have also been reported to affect the composition of the EO [23,25,26,27]. Cuminic aldehyde is basically the main compound of *Cuminum cyminum,* with a concentration of 22.4–41.5% [28,29,30]. On the other hand, *p*-cymen-7-ol was described as a major compound in the EOs of several plants, for instance, *Curcuma* cf. *xanthorrhiza* [31], *Eucalyptus largiflorens* [32].

Diterpenes have been known as rarely described compounds in EOs of the aromatic plants; nevertheless, they were reported as a major compound in the EO of *Lactuca serriola* [33], *Euphorbia mauritanica* [34], *Araucaria bidiwillii* [35], *Araucaria heterophylla* [3,36]. The results of the current study agreed with the scarcity of diterpenes, identifying only one oxygenated diterpenoid, *trans*-geranylgeraniol (0.33%), with a complete absence of diterpene hydrocarbons.

In addition to terpenoid components, four carotenoid-derived compounds were determined in the EO of *K. aegyptiaca* (Table 1). Hexahydrofarnesyl acetone attained a remarkable concentration (14.23%) in the *K. aegyptiaca* (Figure 3). This compound was assessed as an abundant constituent of EOs of *Launaea mucronata* and *Launaea nudicaulis* [37], *H. curassavicum* [23], and *Bassia muricata* [38]. The other hydrocarbons were represented with a relative concentration of 7.67% and ten compounds were represented as a mixture of oxygenated and non-oxygenated compounds. Benzyl acetylacetate with a concentration of 2.32% represented the main non-terpenoids, while *n*-nonadecane (0.27%) represented the minor one. The presence of hydrocarbons in the EO of *K. aegyptiaca* is consistent with the published data of Iranian *K. spuria* [10].

### 2.2. Antioxidant Activity of K. aegyptiaca EO

**Figure 4:**
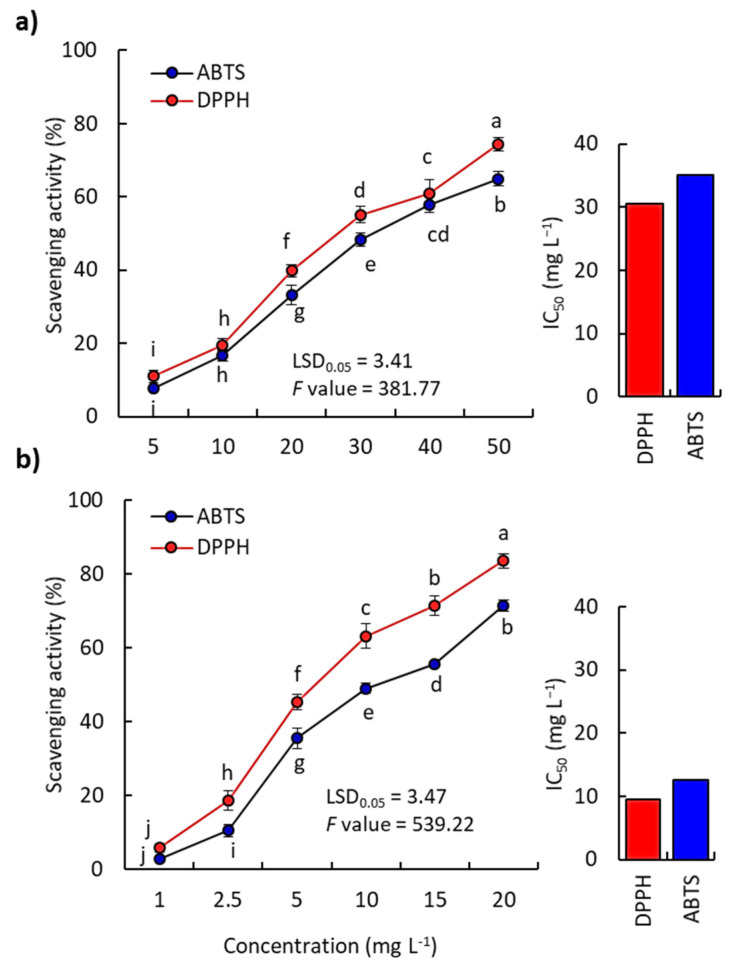
Antioxidant activity of various concentrations and IC_50_ of the essential oil of *K. aegyptiaca* (**a**) and a standard antioxidant, ascorbic acid (**b**) based on the scavenging of DPPH and ABTS. Values are means (*n* = 3) ± standard deviation. Different letters inside each graph reveal values significant variation at *p* ≤ 0.05 (Duncan’s test).

The EO of *K aegyptiaca* showed a substantial antioxidant activity based on both DPPD and ABTS methods compared to the ascorbic acid as a standard synthetic antioxidant (Figure 4). The scavenging activity increased with the increment of EO concentration. At a concentration of 20 mg mL^−1^ of *K. aegyptiaca* EO, the DPPH and ABTS colors were reduced by 39.85% and 33.16%, respectively, while ascorbic acid showed a reduction of 83.48% and 71.33%, respectively, at the same concentration (Figure 4).

According to the IC_50_ data, the *K. aegyptiaca* EO revealed IC_50_ values of 30.48 mg L^−1^ and 35.01 mg L^−1^ for DPPH and ABTS, respectively. The standard antioxidant, ascorbic acid, showed IC_50_ values of 9.45 mg L^−1^ and 12.61 mg L^−1^, regarding DPPH and ABTS, respectively. The considerable antioxidant activity of *K. aegyptiaca* EO in the present study could be attributed to the effect of key compounds, such as cuminic aldehyde, caryophyllene oxide, hexahydrofarnesyl acetone, *ar*-turmerone, aromadendrene oxide, and humulene epoxide. These compounds could act in either singular or in synergistic ways [39,40]. The major compound (cuminic aldehyde) has been reported in a high concentration (52.56%) in *C. cyminum,* showing strong antioxidant activity [29,41]. On the other hand, the second major compound in the present study (caryophyllene oxide) has been reported to possess substantial antioxidant activity [2,42]. The carotenoid-derived compound, hexahydrofarnesyl acetone, has been reported in the EOs of various plants that showed strong antioxidant activity, such as *Launaea* species [37], *H. curassavicum* [23], and *B. muricata* [38]. The aromadendrene oxide-rich EO of *Cleome amblyocarpa* has been described to have allelopathic, antioxidant, and anti-inflammatory activities [43]. The *K. aegyptiaca* EO showed a higher antioxidant activity than the EOs of other reported plants, such as *Persicaria lapathifolia* [25], *Cleome droserifolia* [44], and *Deverra tortuosa* [45], while it showed a lower antioxidant activity than those reported for the EOs of *E. mauritanica* [34].

### 2.3. Antibacterial Activity of K. aegyptiaca EO

The EO extracted from *K. aegyptiaca* aerial parts displayed considerable antibacterial activity against Gram-positive and Gram-negative bacterial isolates (Table 2). The EO showed varying inhibitory activity on various bacterial strains, but it did not reveal antibacterial activity against *Streptococcus epidermis* (Gram-negative strain). The antibacterial effect can be ordered as follows: *Salmonella typhimurium* > *Bacillus cereus* > *Escherichia coli* > *Staphylococcus aureus* > *Pseudomonas aeruginosa* > *Staphylococcus xylosus* > *Staphylococcus haemolyticus* (Table 2).

The selected antibiotics showed varied activity against the bacterial strains, with a general trend that Gram-negative bacteria were more resistant than Gram-positive strains. This observation is consistent with most research [26,45,46,47,48,49], where it is ascribed to the structure of the bacterial cells [46]. Cephradin showed the highest activity against *S. haemolyticus*, while it was inactive against *P. aeruginosa* and *S. typhimurium* at a dose of 10 mg mL^−1^. The tetracycline exhibited maximum inhibition on *S. epidermis*, but did not show activity against *P. aeruginosa*. On the other hand, the antibiotic azithromycin showed maximum activity against *S. aureus*, *S. epidermis*, and *B. cereus* at a concentration of 10 mg mL^−1^, while it did not show any activity against *S. typhimurium.* At a concentration of 10 mg mL^−1^, ampicillin was detected as a powerful antibacterial agent against *S. aureus*, while it did not have any activity against *P. aeruginosa* and *S. typhimurium* (Table 2). Based on the data of the minimum inhibitory concentration (MIC), the EO activity was highest (0.031 mg mL^−1^) againt *E. coli* and *B. cereus*, while the activity against the other bacterial isolates can be sequenced as follows: *S. typhimurium*, *P. aeruginosa*, *S. aureus*, *S. xylosus*, and *S. haemolyticus*. However, *S. epidermis* was comopletely resistant to the EO of *K. aegyptiaca*. The antibacterial activity of the *K. aegyptiaca* EO in the present study was higher than those reported for the EO of *D. tortuosa* [45] and *Teucrium polium* [48], while it was lower than others, such as *Thymus decussatus* [48], *Achillea fragrantissima*, *Artemisia Judaica*, and *Tanacetum sinaicum* [47].

The observed antibacterial activity of *K. aegyptiaca* could be attributed to the activity of the major compounds (cuminic aldehyde, caryophyllene oxide, hexahydrofarnesyl acetone, *ar*-turmerone, aromadendrene oxide, and humulene epoxide), either singly or synergistically. The insecticidal activity of *Rosmarinus officinalis* has been attributed to the synergistic interaction between camphor and 1,8-cineole [40]. A similar study by de Sousa, et al. [50] indicated that the combination of carvacrol and 1,8-cineole maximizes the inhibitory activity against bacterial strains associated with vegetable processing. In addition, cuminic aldehyde has been reported to have antibacterial effects [29,30]. In contrast to our results, the EO of *C. cyminum*, rich with cuminic aldehyde, did not show antibacterial activity against *Pseudomonas* species [29].

Caryophyllene oxide has been reported as a strong antimicrobial agent against a wide range of microbes [51]. In addition, the EOs that were rich in caryophyllene oxide were reported to have a considerable antimicrobial activity, such as *Satureja coerulea* [52], *Psidium guajava* [53], and *Pinus eldarica* [54]. Moreover, EOs rich in hexahydrofarnesyl acetone have been described as possessing considerable antimicrobial activity [55,56]. In addition, *ar*-turmerone has been reported as antimicrobial agent in *Artemisia integrifolia* [57]. Our findings supported the potential uses of the EO of *K. aegyptiaca* in the food industries, as well as in the manufacturing of cosmetics and aromas, due to its potent antioxidant and/or antimicrobial significance.

## 3. Materials and Methods

### 3.1. Plant Materials

**Figure 5:**
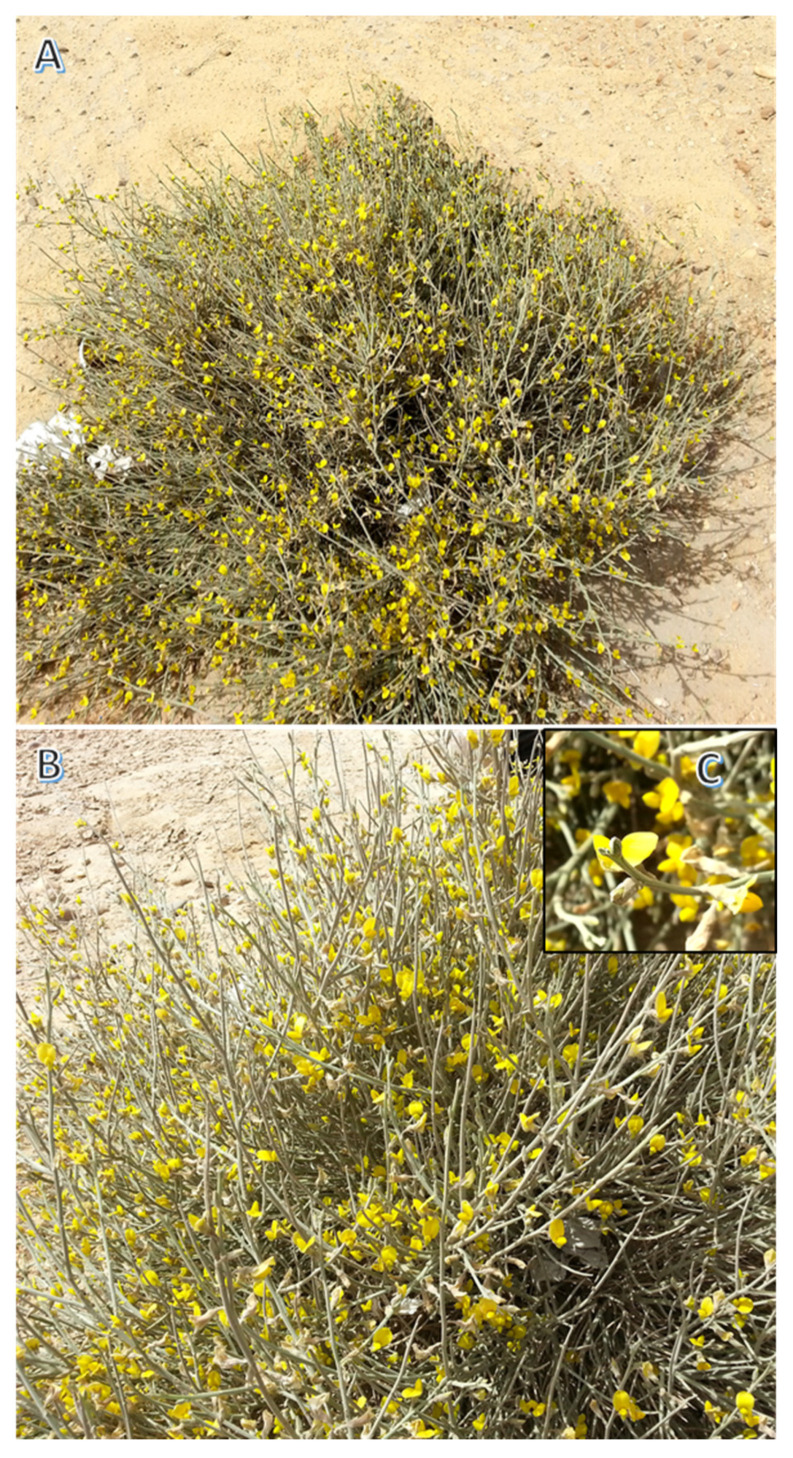
*Kickxia aegyptiaca* (L.) Nábělek. (**A**) overview of the plant in sandy habitat showing dense branching, (**B**) close view showing the flowering branches, and (**C**) close view of the yellow flower.

The *K. aegyptiaca* aerial parts were collected during the flowering season (April 2019) from different populations growing in Wadi Araba, Eastern Desert, Egypt (28.9781482N, 32.2019523E). The collected samples were healthy and flowering. Plant authentication was carried out following Boulos [13] and Tackholm [58]. From the collected sample, a voucher specimen was prepared and deposited in the Botany Department Herbarium at College of Science, Mansoura University, Egypt, with the code Mans.191101007 (Figure 5).

### 3.2. Extraction of EO and GC-MS Analysis

The EO was extracted via the subjection of ~180 g of the air-dried *K. aegyptiaca* aerial parts to hydro-distillation for 3 h over the Clevenger apparatus. The separation of the EO layer was performed by *n*-hexane, dried by anhydrous Na_2_SO_4_ (0.5 g), and then saved at 4 °C in glass vials until further chemical and biological analyses. The extracted EO was chemically analyzed via gas chromatography-mass spectrometry (GC-MS). The characterization and identification of chemical constituents were performed with the same conditions and protocol as described previously [25,59]. The GC-MS apparatus was made up of TRACE GC Ultra-Gas Chromatographs (THERMO Scientific™ Corporate, Waltham, MA, USA) with a quadrupole mass spectrometer (Thermo Scientific ISQ™ EC, Waltham, MA, USA). The GC-MS column dimension was 30 m × 0.32 mm and i.d. 0.25 µm film thickness. At a flow rate of 1.0 mL per min, helium was used as a transporter gas, with a split ratio of 1 to 10. The temperature program was accustomed as follows: 60 °C for 1 min., raised to 240 °C with 4 °C/min. The diluted sample in *n*-hexane (1 µL) at a ratio of 1:10 (*v*/*v*) was injected into the instrument, where the injector and detector were adjusted at 210 °C. The mass spectra of compounds were charted by electron ionization (EI) at 70 eV, using a spectral range of *m*/*z* 40–450. The authentication and identification of the chemical compounds were performed using the Automated Mass spectral Deconvolution and Identification (AMDIS) software, NIST library database, Wiley spectral library collection, retention indices relative to *n*-alkanes (C_8_–C_22_), or assessment of the mass spectrum with authentic standards compounds. The relative concentrations of the compounds were performed based on Tentatively Identified Compounds (TICs) of the EO.

### 3.3. Antioxidant Activity of the EO

To test the antioxidant activity *K. aegyptiaca* EO, two protocols were considered: (a) reduction in the radical 2,2-diphenyl-1-picrylhydrazyl (DPPH, Sigma-Aldrich, Darmstadt, Germany) and (b) reduction in the radical 2,2′-azinobis(3-ethylbenzothiazoline-6-sulfonic acid) (ABTS, Sigma-Aldrich, Darmstadt, Germany). In the DPPH assay, the EO was prepared in a concentration range of 5–50 mg L^−1^, using methanol as a solvent. This range was selected based on the scavenging activity that enabled us to determine the IC_50_ (EO amount necessary to reduce the radical by 50%) [45]. According to Miguel [60], equal volumes of each concentration and DPPH (0.3 mM) were shaken vigorously and kept in dark conditions for 30 min. The absorbance was assessed at 517 nm via spectrophotometer, model Spectronic 21D, Milton Roy, CA, USA. On the other side, the ABTS assay was conducted according to Re, et al. [61]. In brief, about 0.2 mL of each concentration was mixed with 2 mL of freshly prepared ABTS and incubated in a dark condition for 6 min. The range of the EO concentration was similar to those of DPPH (5–50 mg L^−1^). The color absorbance was measured at 734 nm by Spectronic 21D spectrophotometer, Milton Roy, CA, USA. In addition, to refer the antioxidant activity to standard antioxidant, various concentrations (1–20 mg L^−1^) of ascorbic acid were prepared and their antioxidant activity was determined as previously described for the EO. The scavenging activity was calculated based on the following formula:

$$\mathrm{Scavenging}\;\mathrm{activity}\;\left(\%\right)=100\;\times \left(\frac{{\mathrm{Absorbance}}_{\mathrm{sample}\;}{-\;\mathrm{Absorbance}}_{\mathrm{sample}}}{{\mathrm{Absorbance}}_{\mathrm{sample}}}\right)$$

### 3.4. Antibacterial Activity of the EO

The EO extracted from *K. aegyptiaca* aerial parts was tested for its antibacterial activity against four Gram-negative bacterial strains (*E. coli* (ATCC 10536), *P. aeruginosa* (ATCC 9027), *S. typhimurium* (ATCC 25566), and *S. epidermis* (ATCC 12228)) and four Gram-positive bacterial strains (*B. cereus* (EMCC number), *S. aureus* (ATCC 6538), *S. haemolyticus* (ATCC 29970), and *S. xylosus* (NCCP 10937)). The bacterial isolates were obtained from the Cairo Microbiological Resources Centre (Cairo MIRCEN), College of Agriculture, Ain Shams University, Egypt. The bioassay was performed using the agar diffusion method [62]. In brief, filter paper discs (Whatman no.1, 5 mm) were saturated with the EO of *K. aegyptiaca* at a concentration of 10 mg mL^−^^1^ in dimethyl sulfoxide. Petri dishes (90 mm) were prepared and filled with sterilized nutrient agar medium and inoculated with 10^6^ colony-forming units (CFU)/mL of each bacterial strain. The filter paper disc was adjusted above the medium in the center of the Petri dish, and the 1080 plates were immediately sealed with Parafilm^®^ tape (Sigma, St. Louis, MO, USA) and incubated for 24 h at 37 °C. After incubation, the diameter of the inhibition zone (clear zone around disc without growth) was measured in mm at three random positions. The MIC was determined based on the dimensions of the inhibition zone for different EO concentrations. To compare the antibacterial activity of the EO with reference antibiotics, cephradin, tetracycline, azithromycin, and ampicillin were subjected to the same procedures.

### 3.5. Statistical Analysis

The experiments of both antioxidant activity and antibacterial activity were achieved three times with three replications for each treatment. The data were subjected to one-way analysis of variance (ANOVA), after Duncan’s test using CoStat software program (version 6.311, CoHort Software, Monterey, CA, USA). The data were expressed as mean values with standard error. The IC_50_ value for antioxidant assays was graphically calculated using MS-Excel 2016.

## 4. Conclusions

A GC-MS analysis of the extracted EO from the aerial parts of *K. aegyptiaca* revealed, for the first time, the presence of 43 compounds, mainly terpenes. Oxygenated compounds were predominant, particularly sesquiterpenes and monoterpenes. Cuminic aldehyde, caryophyllene oxide, hexahydrofarnesyl acetone, *ar*-turmerone, aromadendrene oxide, and humulene epoxide were identified as major compounds with a concentration of 66.02% of the total mass. The extracted EO showed considerable antioxidant activity as well as antibacterial activity. The major compounds have been reported to possess various biological activities, including antioxidant and antimicrobial activities. Therefore, further studies are recommended to evaluate the various biological activities of the major identified compounds, either alone or in combination, as well as to assess their biosafety.

## Figures and Tables

**Table 1:** Chemical profile characterization of the essential oil extracted from the aerial parts of *K. aegyptiaca*.

No	Rt ^a^	Conc.% ^b^	Compound	Formula	KI ^c^
	Oxygenated monoterpenes				
1	6.39	1.31 ± 0.03	*α*-Terpineol	C_10_H_18_O	1186
2	9.82	0.55 ± 0.01	*α*-Linalool	C_10_H_18_O	1095
3	11.92	21.99 ± 0.21	Cuminic aldehyde	C_10_H_12_O	1239
4	13.42	2.17 ± 0.06	*p*-Cymen-7-ol	C_10_H_14_O	1287
5	13.87	1.45 ± 0.04	Carvacrol	C_10_H_14_O	1298
6	16.07	1.51 ± 0.04	Eugenol	C_10_H_12_O_2_	1356
7	17.26	0.84 ± 0.02	10-(acetylmethyl)-3-Carene	C_13_H_20_O	1380
	Monoterpene hydrocarbons				
8	10.82	0.63 ± 0.03	2-Bornene	C_10_H_16_	1165
	Oxygenated sesquiterpenes				
9	19.68	0.65 ± 0.03	Neryl acetone	C_13_H_22_O	1436
10	21.81	0.25 ± 0.01	trans-Nerolidol	C_15_H_26_O	1561
11	24.02	0.43 ± 0.01	trans-Sesquisabinene hydrate	C_15_H_26_O	1577
12	24.16	0.97 ± 0.02	Spathulenol	C_15_H_24_O	1577
13	25.38	0.95 ± 0.02	Isoaromadendrene epoxide	C_15_H_24_O	1579
14	24.59	17.34 ± 0.11	Caryophyllene oxide	C_15_H_24_O	1582
15	25.19	0.42 ± 0.01	Carotol	C_15_H_26_O	1594
16	25.58	1.24 ± 0.03	Widdrol	C_15_H_26_O	1599
17	26.8	2.70 ± 0.06	Humulene epoxide	C_15_H_24_O	1608
18	27.05	0.43 ± 0.03	Clov-2-ene-9α-ol	C_15_H_24_O	1616
19	27.53	3.84 ± 0.07	Aromadendrene oxide-(2)	C_15_H_24_O	1631
20	28.14	8.51 ± 0.20	ar-Turmerone	C_15_H_20_O	1669
22	34.21	1.85 ± 0.04	*trans*-*Z*-*α*-Bisabolene epoxide	C_15_H_24_O	1675
22	36.28	0.53 ± 0.02	(*E*,*E*)-Farnesyl acetone	C_18_H_30_O	1915
	Sesquiterpene hydrocarbons				
23	17.72	1.07 ± 0.03	Longicyclene	C_15_H_24_	1374
24	18.24	1.97 ± 0.04	Isocaryophillene	C_15_H_24_	1408
25	19.42	0.39 ± 0.02	trans-Caryophyllene	C_15_H_24_	1417
26	20.32	0.57 ± 0.01	*β*-Farnesene	C_15_H_24_	1442
27	20.53	0.31 ± 0.01	ar-Curcumene	C_15_H_22_	1480
28	22.85	0.26 ± 0.01	*α*-Calacorene	C_15_H_20_	1544
	Oxygenated diterpenes				
29	29.86	0.33 ± 0.02	*trans*-Geranylgeraniol	C_20_H_34_O	2201
	Carotenoid derived compounds				
30	12.77	1.21 ± 0.04	dihydroedulan II	C_13_H_22_O	1284
31	13.13	0.56 ± 0.02	Theaspirane A	C_13_H_22_O	1298
32	16.84	0.72 ± 0.03	*β*-Damascenone	C_13_H_18_O	1384
33	34.02	11.74 ± 0.13	Hexahydrofarnesyl acetone	C_18_H_36_O	1845
	Others				
34	28.24	2.32 ± 0.06	Benzyl acetylacetate	C_11_H_12_O_3_	1486
35	32.11	0.30 ± 0.01	*n*-Octadecyl chloride	C_18_H_57_Cl	1399
36	35.48	0.27 ± 0.01	*n*-Nonadecane	C_17_H_34_O_2_	1900
37	36.77	0.78 ± 0.02	Methyl palmitate	C_21_H_44_	1921
38	41.77	0.93 ± 0.03	*n*-Heneicosane	C_17_H_34_O_2_	2100
39	43.03	0.50 ± 0.02	9,12-Octadecadienoic acid	C_18_H_32_O_2_	2085
40	44.69	0.26 ± 0.01	2-Nonadecanone	C_19_H_38_O	2106
41	47.52	0.78 ± 0.02	*n*-Docosane	C_22_H_46_	2200
42	48.14	0.54 ± 0.01	*n*-Tetracosane	C_24_H_50_	2400
43	57.76	0.99 ± 0.04	*n*-Octacosane	C_28_H_58_	2800
Total		97.36			

^a^ retention time, ^b^ average concentration of three replications ± standard deviation, ^c^ Kovats retention index.

**Table 2:** Antibacterial activity of the essential oil extracted from *K. aegyptiaca* aerial parts, expressed by the diameter of the inhibition zone (mm) and minimum inhibitory concentration (MIC), as well as some selected reference antibiotics at a concentration of 10 mg mL^−1^.

Microbes	*K. aegyptiaca* (10 mg mL^−1^)	MIC (10 mg mL^−1^)	Cephradin	Tetracycline	Azithromycin	Ampicillin
Gram-negative bacteria						
*Escherichia coli*	22.04 ± 0.74 ^C,^#	0.031	15.67 ± 0.42 ^E^	20.11 ± 0.55 ^B^	18.08 ± 0.44 ^C^	20.97 ± 0.75 ^C^
*Pseudomonas aeruginosa*	13.67 ± 0.91 ^E^	0.044	0.00 ^G^	0.00 ^E^	12.57 ± 0.31 ^D^	0.00 ^F^
*Salmonella typhimurium*	26.08 ± 1.02 ^A^	0.038	0.00 ^G^	9.47 ± 0.37 ^D^	0.00 ^E^	0.00 ^F^
*Streptococcus epidermis*	0.00 ^H^	0.00	11.05 ± 0.81 ^F^	21.07 ± 0.98 ^A^	20.36 ± 0.77 ^A^	10.57 ± 0.57 ^D^
Gram-positive bacteria						
*Bacillus cereus*	23.11 ± 0.58 ^B^	0.031	19.6 ± 0.43 ^C^	9.68 ± 0.27 ^D^	20.15 ± 0.33 ^A^	6.45 ± 0.36 ^E^
*Staphylococcus aureus*	16.17 ± 0.51 ^D^	0.052	20.17 ± 0.79 ^B^	18.51 ± 0.65 ^C^	20.48 ± 0.49 ^A^	29.14 ± 1.20 ^A^
*Staphylococcus haemolyticus*	6.24 ± 0.11 ^G^	0.562	24.17 ± 0.66 ^A^	20.30 ± 1.01 ^B^	19.19 ± 0.61 ^B^	20.95 ± 0.94 ^C^
*Staphylococcus xylosus*	11.61 ± 0.32 ^F^	0.092	18.34 ± 0.77 ^D^	18.48 ± 0.88 ^C^	18.75 ± 0.73 ^B^	24.66 ± 0.68 ^B^
*LSD*_0.05_	0.51 ***		0.52 ***	0.49 ***	0.45 ***	0.44 ***

# values are average (*n* = 3) ± standard error. Dissimilar superscript letters in each treatment express significant variation at a probability level of 0.05 (Duncan’s test). LSD: least significant difference. *** *p* < 0.001.

## Data Availability

All figures and Tables are original.

## Sample Availability

Samples of the compounds are not available from the authors.
